# Lifestyle Changes and Determinants of Children’s and Adolescents’ Body Weight Increase during the First COVID-19 Lockdown in Greece: The COV-EAT Study

**DOI:** 10.3390/nu13030930

**Published:** 2021-03-13

**Authors:** Odysseas Androutsos, Maria Perperidi, Christos Georgiou, Giorgos Chouliaras

**Affiliations:** 1 Department of Nutrition and Dietetics, School of Physical Education, Sport Science and Dietetics, University of Thessaly, 42132 Trikala, Greece; mperper@msn.com (M.P.); cri.georgiou@gmail.com (C.G.); 2 Second Department of Paediatrics, School of Medicine, National and Kapodistrian University of Athens, 11527 Athens, Greece; georgehouliaras@msn.com

**Keywords:** COVID-19, obesity, children, lifestyle, determinants, diet, physical activity, sedentary behavior, COV-EAT

## Abstract

Previous studies showed that the coronavirus disease 2019 (COVID-19) lockdown imposed changes in adults’ lifestyle behaviors; however, there is limited information regarding the effects on youth. The COV-EAT study aimed to report changes in children’s and adolescents’ lifestyle habits during the first COVID-19 lockdown and explore potential associations between changes of participants’ lifestyle behaviors and body weight. An online survey among 397 children/adolescents and their parents across 63 municipalities in Greece was conducted in April–May 2020. Parents self-reported changes of their children’s lifestyle habits and body weight, as well as sociodemographic data of their family. The present study shows that during the lockdown, children’s/adolescents’ sleep duration and screen time increased, while their physical activity decreased. Their consumption of fruits and fresh fruit juices, vegetables, dairy products, pasta, sweets, total snacks, and breakfast increased, while fast-food consumption decreased. Body weight increased in 35% of children/adolescents. A multiple regression analysis showed that the body weight increase was associated with increased consumption of breakfast, salty snacks, and total snacks and with decreased physical activity. The COV-EAT study revealed changes in children’s and adolescents’ lifestyle behaviors during the first COVID-19 lockdown in Greece. Effective strategies are needed to prevent excessive body weight gain in future COVID-19 lockdowns.

## 1. Introduction

Since December 2019, the world is facing a new disease (coronavirus disease 2019 (COVID-19)), caused by the severe acute respiratory syndrome coronavirus 2 (SARS-CoV-2). The WHO declared COVID-19 a pandemic in March 2020. In Greece, a number of regulatory measures have been implemented, including the closing of schools on 11 March and finally a lockdown, imposed on 23 March as a “last-resort” preventive measure to halt the spreading of the disease until COVID-19 vaccines would be available. This unprecedented situation led to significant changes in children’s daily routine, who no longer attended school and out-of-school activities (e.g., participation in sports, free play at playgrounds, etc.), but were isolated at home with their families.

Studies conducted during the COVID-19 pandemic in adults have shown that self-isolation at home due to the lockdown was associated with lower level of physical activity, longer sedentary time, modifications in eating behavior, and sleeping disturbances [1,2,3]. Furthermore, an increase of food purchased before the pandemic was reported in some countries, which increased the availability of foods during the lockdown [4]. Self-isolation has been also linked to boredom and stress, which in turn may lead to higher energy intake, consumption of energy-dense “comfort foods”, and emotional eating [5]. To date, there is a lack of studies focusing on changes of children’s lifestyle behaviors during the COVID-19 lockdown. However, there are studies which have examined the impact of school closing in the summer period, which is a condition similar to the COVID-19 lockdown, on children’s eating behavior and weight status [3,6,7,8,9]. The majority of these studies concluded that children’s body mass index (BMI) increased more rapidly during summer vacation compared to school period [7]. Moreover, it was revealed that children tend to consume less vegetables and more sugar, spend more time on TV-watching, and be more active during summer breaks compared to school time [8]. Interestingly, the study of Von Hippel et al. showed that the prevalence of childhood obesity increased only during summer vacations and not during any other time period of the year [6].

Factors that may influence children’s eating behavior have been previously reported and include certain lifestyle behaviors, such as sedentary behavior [10,11,12,13,14,15], insufficient sleep duration [10,12,13,14], and inactivity [10,11,12,13,15,16,17], as well as determinants from children’s social and physical environment, such as parental modeling [10,11,16,17] and availability of foods at home [11,16]. It is also known that retaining higher energy intake compared to energy expenditure for long periods of time increases the risk of overweight/obesity [18]. 

The scientific community raises concerns about the health implications that may be caused by the COVID-19 lockdown [1,2,19,20,21,22]. The present study aims to report changes in children’s and adolescents’ lifestyle behaviors during the first lockdown that was implemented in Greece due to COVID-19 and explore potential associations with changes of their body weight.

## 2. Materials and Methods

### 2.1. Study Design and Participants

The COV-EAT study was a cross-sectional study, which was conducted across 63 municipalities in Greece. Parents having children aged 2–18 years were invited to participate. The following inclusion criteria were applied: living in Greece, being able to complete the study questionnaire in Greek language, having children aged 2–18 years, and providing a consent form. The COV-EAT study adhered to the Declaration of Helsinki and the conventions of the Council of Europe on human rights and Biomedicine. The study protocol was approved by the Ethical Committee of the Department of Physical Education and Sport Science in the School of Physical Education, Sport Science, and Dietetics, University of Thessaly, and registered at clinicaltrials.org (NCT04437121). All parents electronically signed an informed consent form prior to their participation in the study. Only one child per family was included in this study.

### 2.2. Instruments and Variables

An online survey was conducted in a sample of families, who were invited to electronically fill in a questionnaire which included 70 questions divided in 3 sections. The first section focused on participants’ sociodemographic data and included 15 questions about child’s gender, parents’ and child’s age, number of children in the family, city of residence, and socioeconomic status. The second section contained 20 questions about parents’ dietary and lifestyle habits before and during the lockdown. Specifically, parents had to answer questions about the frequency of cooking at home and their fast-food consumption, the number of meals and snacks consumed per day, the reasons of snacking, and their body weight and height. The third section contained 35 questions about their child’s eating habits and its sedentary behavior. Parents were asked to report their child’s body weight and height, the number of meals and snacks during the day, the frequency of breakfast, fast-food, fruits, juices, vegetables, dairy, red meat, poultry, fish, pasta, legumes, sweets, salty snacks, and beverages consumption, the servings of fruits, juices, vegetables, and dairy consumed per day, vitamin supplementation, as well as screen time, sleep duration, and changes in physical activity. All answers were given about the period before and during the lockdown. According to the difference between the values of each lifestyle behavior before and during the lockdown, each parameter was categorized as “decrease” (i.e., before-lockdown value of lifestyle behavior higher than during-lockdown value), “stable” (i.e., same values of lifestyle behavior before and during the lockdown), or “increase” (i.e., before-lockdown value of lifestyle behavior lower than during-lockdown value).

The questionnaire is available as a Supplementary Materials.

### 2.3. Procedure

All data were collected between 30 April and 24 May 2020. The questionnaire was created using the Google Forms tool and was distributed electronically via networks of dietitians/nutritionists in Greece, personal networks, and social media.

### 2.4. Statistical Analyses

Continuous data are presented as mean ± standard deviation (SD). Categorical variables are presented as absolute (n) and relative (%) frequencies. Mean values of consumption of food groups before and during the quarantine were compared using the Student’s test for paired data. Associations between categorical data were evaluated with Fisher’s exact test. For paired pre- vs. post-comparisons of sleeping time, the extended McNemar’s test (allowing for 3 × 3 contingency tables in matched observations) was used. For paired pre- vs. post-comparisons of screen time, the Wilcoxon matched-pairs signed-rank test was applied. A stepwise, backwards, regression approach was utilized to assess the effect of dietary data on the probability of body weight increase versus no change or decrease (logistic regression). Participants that answered “don’t know” were treated as missing values. The initial, full model included dietary data, gender, age, area of residency, change in sleep and screen time, and change in physical activity. For logistic regression analysis, results are reported as odds ratios, along with 95% confidence intervals (CI) and *p*-values. The level of statistical significance was set to 0.05 for analyses, and in cases of multiple comparisons, the Bonferroni correction was applied. All analyses were run in Stata 11 MP statistical software (StataCorp., College Station, TX, USA).

## 3. Results

In total, 397 dyads of children/adolescents (51.4% boys) with an average age of 7.8 (4.1) years were recruited. Families’ characteristics are presented in Table 1, and detailed demographic and socioeconomic data are presented in Table 2.

Table 3 and Table 4 present children’s/adolescents’ changes of lifestyle behaviors before vs. during the lockdown. Specifically, during the lockdown, more children/adolescents tended to sleep longer than 10 h/night, and fewer slept less than 8 h/night than before the lockdown. Similarly, the children/adolescents who spent more than 3 h/day in front of a screen were more during home isolation, whereas those who did not spent time on a screen were less during the confinement than before the confinement. Moreover, 66.9% of the parents reported that their child’s physical activity level was decreased during the lockdown, and 35% that their child’s body weight increased. Regarding children’s eating behavior, the consumption of fruits and fresh fruit juices, vegetables, dairy products, pasta, sweets, total snacks, and breakfast significantly increased (*p* < 0.05). In contrast, fast-food consumption was significantly decreased (*p* < 0.001). No significant changes were observed in core foods used for lunch and dinner, such as red meat, poultry, fish, and legumes. Similarly, no significant changes were observed in the consumption of prepacked juices and sodas and salty snacks.

Table 5 presents the bivariate correlations between body weight increase and changes in lifestyle behaviors. More specifically, body weight increase was correlated with increase of consumption of salty snacks and red meat (*p* < 0.05). Similarly, increase of sleep duration (*p* = 0.012) and screen time (*p* < 0.001) and decrease of physical activity (*p* < 0.001) were associated with body weight increase. No significant associations were observed between body weight change and consumption of fresh fruits and fruit juices, vegetables, poultry, fish, pasta, and legumes.

The results of a multiple, stepwise, backwards, logistic regression analysis of the associations between children’s and adolescents’ body weight increase and changes of their lifestyle behaviors during the lockdown are shown in Table 6. Based on these findings, increase of consumption of breakfast, salty snacks, and total snacks along with decrease of physical activity were significantly associated with increase of children’s and adolescents’ body weight.

## 4. Discussion

The COV-EAT study is the first study in Greece and one of the few globally that examined changes of lifestyle behaviors in children and adolescents during the first lockdown that was implemented due to COVID-19 and explored their associations with children’s/adolescents’ body weight gain. The main findings of the present study showed that during the lockdown period, children and adolescents in Greece: (1) increased their consumption of certain foods, such as fruits and fresh juices, vegetables, dairy, pasta, sweets, total snacks, and decreased their fast-food consumption, (2) increased their screen time, (3) increased their sleep duration, (4) decreased their physical activity, and (5) 35% of them gained body weight. According to the results of a multiple regression analysis conducted in this study, increased consumption of breakfast, salty snacks, and total snacks along with decreased physical activity was significantly associated with increase of children’s and adolescents’ body weight.

The findings of the present study suggest that the COVID-19 lockdown, with the concomitant closure of schools, negatively affected children’s lifestyle behaviors, which are some of the predominant risk factors for obesity [3]. In line with the findings of the current study, Pietrobelli et al. showed that during the lockdown, children and adolescents with obesity in Italy significantly increased their consumption of certain foods (chips, red meat, and sugary drinks), their sleep duration, and the time they devoted to screen activities, while they decreased the time they spent in sports [3]. Similarly, Ng et al. in a sample of 1214 Irish adolescents, showed that half of the participants tended to decrease their physical activity during the lockdown, especially those with overweight or obesity [23]. Furthermore, Jia et al. conducted a survey among 10,082 participants from high schools, colleges, and graduate schools (aged 19.8 ± 2.3 years) and showed that individuals’ BMI, screen time, and sedentary and sleeping time on weekdays and weekends increased, while the frequency of engaging in active transport, moderate/vigorous-intensity housework, leisure-time moderate/vigorous-intensity physical activity, and leisure-time walking were decreased [24]. Also, the study by Ruiz-Roso et al. in a multinational sample of adolescents from Italy, Spain, Chile, Colombia, and Brazil indicated that families had more time to cook and improved eating habits by increasing legumes, vegetables and fruits intake and reducing fast-food consumption, but that was not enough to increase the overall diet quality, because of the higher sweet food and fried food consumption [25]. Similarly, in our study, fast-food consumption decreased (*p* < 0.001), which might have resulted from the fear of being affected by the coronavirus that could be transmitted from the person delivering the food. The COV-EAT study was conducted during the first quarantine, where ignorance of the protective measures against COVID-19 was excessive. Studies in adults are also in line with the findings of the COV-EAT study. According to the preliminary results of the ECLB-COVID19 international study in 1047 adults, the COVID-19 lockdown had a negative effect on physical activity and eating behavior and led to a significant increase in sitting time [26].

The alterations in children’s and adolescent’s lifestyle behaviors may be explained in different ways. The decrease of physical activity may be attributed to home confinement, which does not allow individuals to attend sport clubs and organized physical activity or visit schoolyards, parks, and recreational areas. Ng et al. reported that Irish adolescents with overweight were more likely to be less physically active during the COVID-19 lockdown [23]. In contrast, the increase of screen time may be due to the longer duration of distance learning replacing both school lessons and private lessons, in addition to more free time at home and to boredom. The increase of sleep duration may be linked to the fact that children did not have to go to school in the morning. Changes of eating behavior may be caused by several different factors. First, the insecurity caused by COVID-19 may have led families to change the home food environment and feeding practices. Indeed, Adams et al. reported that families experiencing food insecurity exerted greater pressure to their children to eat, while 30% of the families increased the amount of high-calorie snack foods, desserts/sweets, and fresh foods, and almost half of the study sample increased the availability of non-perishable processed foods in their homes [27]. In addition to the physical environment, the social environment at home may have changed because of COVID-19. In the present study, a number of parents (5.1% of mothers and 2.9% of fathers) lost their job, while others (4.2% of mothers and 3.2% of fathers) experienced an increase of working time during the COVID-19 lockdown. These changes might be associated with parental stress and disturbances of family interactions, parental modeling, and parenting feeding practices at home [28]. Furthermore, the psychological impact of self-isolation may have triggered boredom and stress, which are determinants of the consumption of energy-dense “comfort foods” and emotional eating [5]. Especially children and adolescents with obesity may be more susceptible to overeating, as observed in Polish adults with overweight and obesity [29]. It is also noted that cooking and preparation of new recipes for snacks might be used as a recreational activity by the family during the lockdown, which in turn increases the availability of home-made sweets, snacks, and foods. Additionally, a home does not always provide a steady environment for mealtimes, physical activity, and sleep schedule [3].

Changes of lifestyle behaviors may lead to an increase of energy intake over energy expenditure, a condition which results in body weight gain when lasting for long periods of time. As expected, increased consumption of energy-dense foods (i.e., total snacks, including sweets) and decreased physical activity were associated with an increase of children’s and adolescents’ body weight. Still, the present study also showed that the increases of breakfast consumption and total snacks were associated with an increase of participants’ body weight. These observations may be attributed to the fact that children/adolescents increased the number of meals consumed per day and that, possibly, they consumed unhealthy foods/snacks in these extra meals. Future studies should shed more light on these associations. Moreover, increased body weight in 35% of children and adolescents could be a natural trend due to children’s growth if the increase of body weight was about 0.5 kg. The mean body weight increase of 2 kg indicates an abnormal weight gain. 

Since obesity and its complications (diabetes, heart disease, pulmonary disease, hypertension, etc.) can worsen the implications of COVID-19, it is critical to implement measures, during the lockdown, to promote healthy eating and physical activity and prevent obesity [3,9,19,30,31,32]. To achieve these goals, an umbrella of telehealth (e-health and m-health) obesity prevention and treatment actions should be implemented, in addition to the measures taken to tackle the expansion of COVID-19 [30,33]. Policy interventions to oversee food advertisements and behavioral strategies to promote nutrition education, appetite control, and family meal planning should be applied. Vulnerable groups, such as children and adolescents with overweight or obesity, lower socioeconomic groups, and families with food insecurity should be prioritized. 

The findings of the current study should be interpreted under the light of its strengths and limitations. Regarding the strengths, the COV-EAT study was the first study in Greece to explore the potential effect of the first COVID-19 lockdown in Greece on children’s and adolescents’ lifestyle behaviors, using data from 397 families from urban, semi-urban, and rural areas. Regarding the limitations, firstly due to its cross-sectional design, no causal relationships could be established. Secondly, the study sample was not representative of all children and adolescents in Greece; therefore, the results cannot be generalized to the whole population of Greek children and adolescents. The sampling procedure, which was based on an online survey, may have also produced a selection bias regarding the recruited participants. Moreover, the questionnaire used in the COV-EAT study was not validated, while data were self-reported, and thus subject to recall bias and socially desirable answers, and only weight change was reported. It was also not feasible to conduct comparisons between maternal and paternal reports, which may have an effect on the results. Still, 90% of the reports were taken from mothers, which limits the possibility of such bias.

## 5. Conclusions

The COV-EAT study reported unfavorable changes in children’s and adolescents’ lifestyle behaviors during the first COVID-19 lockdown that was implemented in Greece in spring 2020. Certain lifestyle changes were associated with children’s/adolescents’ body weight gain. Considering that the COVID-19 pandemic may lead to further lockdowns, effective e-health and m-health strategies and programs to tackle the adoption of unhealthy lifestyle behaviors and prevent excessive body weight gain are urgently needed.

## Figures and Tables

**Table 1:** Families’ age and anthropometric characteristics. The COV-EAT study.

Characteristics	Mean ± SD (*n* = 397)
Children’s/adolescents’ age (years)	7.8 ± 4.1
Children’s/adolescents’ body weight (Kg)	32.3 ± 16.9
Fathers’ age (years)	43.2 ± 6.4
Fathers’ body weight (Kg)	88.7 ± 12.9
Fathers’ body height (cm)	179.2 ± 6.3
Mothers’ age (years)	39.8 ± 5.3
Mothers’ body weight (Kg)	70.1 ± 14.1
Mothers’ body height (cm)	165.5 ± 5.9

**Table 2:** Family socio-demographic status. The COV-EAT study.

Variables		N (%)
Area of residence	Urban	331 (83.4%)
	Semi-urban	35 (8.8%)
	Rural	31 (7.8%)
Parental marital status	Core families—married parents	370 (93.2%)
	Single-parent families	27 (6.8%)
Fathers’ occupation before lockdown	Employee	378 (95.2%)
	Unemployed	11 (2.8%)
	Retired	8 (2.0%)
Fathers who lost their job during lockdown		11 (2.9%)
Fathers who increased their working hours during the lockdown		12 (3.2%)
Mothers’ occupation before lockdown	Employee	311 (78.3%)
	Unemployed	84 (21.2%)
	Retired	2 (0.5%)
Mothers who lost their job during lockdown		16 (5.1%)
Mothers who increased their working hours during the lockdown		13 (4.2%)

Data are shown as absolute (*n*) and relative (%) frequencies. Missing values were not included in the statistical analyses.

**Table 3:** Changes of children’s and adolescents’ lifestyle habits * and body weight in the first coronavirus disease 2019 (COVID-19) lockdown in Greece. The COV-EAT study.

Lifestyle Habits		Before Lockdown		During Lockdown	
		Sample (*n* = 397)	Sample (%)	Sample (*n* = 397)	Sample (%)
Sleep duration before and during the first COVID-19 lockdown **	<8 h	61	15.4%	19	4.8%
	8–10 h	283	71.3%	282	71.0%
	>10 h	53	13.3%	96	24.2%
Screen time before and during the first COVID-19 lockdown ***	0 h	19	4.8%	4	1.0%
	1 h	135	34.0%	43	10.8%
	2 h	150	37.8%	79	19.9%
	3 h	66	16.6%	121	30.5%
	>3 h	27	6.8%	150	37.8%
Physical activity change during the COVID-19 lockdown ****	No Change			71	18.2%
	Increase			58	14.9%
	Decrease			261	66.9%
Body weight change during the COVID-19 lockdown *****	No Change			214	58.9%
	Increase			127	35%
	Decrease			22	6.1%

* Data are shown as absolute (*n*) and relative (%) frequencies. ** Extended McNemar’s test: *p* < 0.001. *** Wilcoxon matched-pairs signed-rank test: *p* < 0.001. **** Seven missing values for the variable (1.8% “don’t know” responses of the total). ***** Thirty-four missing values for the variable (8.6% “don’t know” responses of the total).

**Table 4:** Changes of children’s * and adolescents’ eating habits in the first COVID-19 lockdown in Greece. The COV-EAT study.

Food Groups	Before Lockdown	During Lockdown	*p*-Value **
Salty snacks	0.18 (0.10)	0.19 (0.11)	0.12
Fruits and Fresh juices	1.80 (1.21)	2.08 (1.39)	<0.001
Vegetables	0.69 (0.73)	0.76 (0.77)	<0.001
Prepacked juices and sodas	0.26 (0.51)	0.28 (0.51)	0.33
Dairy	1.80 (0.93)	1.92 (0.97)	<0.001
Red meat	0.29 (0.16)	0.29 (0.16)	0.57
Poultry	0.23 (0.11)	0.23 (0.11)	0.64
Fish	0.15 (0.09)	0.15 (0.10)	0.26
Pasta	0.47 (0.27)	0.48 (0.27)	0.014
Legumes	0.20 (0.10)	0.20 (0.11)	0.57
Sweets	0.65 (0.24)	0.73 (0.24)	<0.001
Total snacks	1.95 (0.67)	2.41 (0.86)	<0.001
Fast-food	0.13 (0.13)	0.08 (1.43)	<0.001
Breakfast	6.13 (1.85)	6.56 (1.33)	<0.001

* Data presented as mean (SD) consumption (in servings/day) of each food. Breakfast consumption indicates the frequency of consumption on a weekly level. ** *t*-test for paired data/students test.

**Table 5:** Associations between dietary or lifestyle changes and children’s and adolescents’ body weight (BW) changes during the first COVID-19 lockdown in Greece. The COV-EAT study.

BW Change	Lifestyle Determinants			*p*-Value
	Decrease **	Stable **	Increase **	
Salty snack consumption				
Non-increased BW * (*n* = 236)	27 (77.1%)	199 (70.1%)	10 (22.7%)	<0.001
Increased BW (*n* = 127)	8 (22.9%)	85 (29.9%)	34 (77.3%)	
Fruits and fresh juices				
Non-increased BW * (*n* = 236)	33 (60.0%)	124 (68.9%)	79 (61.7%)	0.3
Increased BW (*n* = 127)	22 (40.0%)	56 (31.1%)	49 (38.3%)	
Vegetables Consumption				
Non-increased BW * (*n* = 236)	10 (52.6%)	192 (66.4%)	34 (61.8%)	0.4
Increased BW (*n* = 127)	9 (47.4%)	97 (33.6%)	21 (38.2%)	
Prepacked juiced and sodas				
Non-increased BW * (*n* = 236)	30 (60.0%)	182 (70.3%)	24 (44.4%)	<0.001
Increased BW (*n* = 127)	20 (40.0%)	77 (29.7%)	30 (55.6%)	
Dairy Consumption				
Non-increased BW * (*n* = 236)	10 (55.6%)	199 (69.1%)	27 (47.4%)	0.005
Increased BW (*n* = 127)	8 (44.4%)	89 (30.9%)	30 (52.6%)	
Red meat Consumption				
Non-increased BW * (*n* = 236)	13 (72.2%)	215 (66.6%)	8 (36.4%)	0.016
Increased BW (*n* = 127)	5 (27.8%)	108 (33.4%)	14 (63.6%)	
Poultry Consumption				
Non-increased BW * (*n* = 236)	8 (80.0%)	221 (64.8%)	7 (58.3%)	0.6
Increased BW (*n* = 127)	2 (20.0%)	120 (35.2%)	5 (41.7%)	
Fish Consumption				
Non-increased BW * (*n* = 236)	12 (80.0%)	214 (65.2%)	10 (50.0%)	0.2
Increased BW (*n* = 127)	3 (20.0%)	114 (34.8%)	10 (50.0%)	
Pasta Consumption				
Non-increased BW * (*n* = 236)	7 (58.3%)	216 (66.3%)	13 (52.0%)	0.3
Increased BW (*n* = 127)	5 (41.7%)	110 (33.7%)	12 (48.0%)	
Legumes Consumption				
Non-increased BW * (*n* = 236)	8 (61.5%)	218 (65.5%)	10 (58.8%)	0.8
Increased BW (*n* = 127)	5 (38.5%)	115 (34.5%)	7 (41.2%)	
Sweets Consumption				
Non-increased BW * (*n* = 236)	26 (65.0%)	158 (72.5%)	52 (49.5%)	<0.001
Increased BW (*n* = 127)	14 (35.0%)	60 (27.5%)	53 (50.5%)	
Total Snacks Consumption				
Non-increased BW * (*n* = 236)	12 (63.2%)	159 (80.7%)	65 (44.2%)	<0.001
Increased BW (*n* = 127)	7 (36.8%)	38 (19.3%)	82 (55.8%)	
Fast-food Frequency				
Non-increased BW * (*n* = 236)	100 (62.5%)	128 (69.6%)	8 (42.1%)	0.043
Increased BW (*n* = 127)	60 (37.5%)	56 (30.4%)	11 (57.9%)	
Breakfast Frequency				
Non-increased BW * (*n* = 236)	10 (47.6%)	200 (69.9%)	26 (46.4%)	0.001
Increased BW (*n* = 127)	11 (52.4%)	86 (30.1%)	30 (53.6%)	
Sleep Duration				
Stable BW	12 (60.0%)	176 (69.8%)	48 (52.7%)	0.012
Increased BW	8 (40.0%)	76 (30.2%)	43 (47.3%)	
Screen Time				
Stable BW	2 (40.0%)	83 (79.1%)	151 (59.7%)	<0.001
Increased BW	3 (60.0%)	22 (20.9%)	102 (40.3%)	
Physical Activity Changes				
Stable BW	132 (55.0%)	59 (89.4%)	43 (79.6%)	<0.001
Increased BW	108 (45.0%)	7 (10.6%)	11 (20.4%)	

Data are shown as absolute (n) and relative (%) frequencies. * Non-increased body weight means either stable or decreased. ** According to the difference between the values of each lifestyle behavior before and during lockdown, they were categorized as “decrease”, “stable, or “increase”.

**Table 6:** Multiple regression analysis between the probability of children’s and adolescent’s body weight increase and dietary and lifestyle changes during the first COVID-19 lockdown in Greece. The COV-EAT study.

		OR	95% CI	*p*-Values
**Breakfast frequency**	Increase vs. No Change	2.3	1.8–4.4	0.015
	Increase vs. Decrease	1.7	0.5–1.5	0.359
**Salty Snacks Consumption**	Increase vs. No Change	4.2	1.9–9.3	0.001
	Increase vs. Decrease	6.7	2.1–20.9	0.001
**Total Snack Consumption**	Increase vs. No Change	3.2	1.9–5.4	<0.001
	Increase vs. Decrease	1.6	0.5–4.7	0.399
**Physical Activity**	No change vs. increase	2.4	0.8–7.1	0.116
	Decrease vs. Increase	5.1	2.1–12.2	<0.001

Data are shown as odds ratios (OR), along with 95% confidence intervals (95% CI) and *p*-values.

## Data Availability

The data presented in this study are available on request from the corresponding author. The data are not publicly available due to ethical restrictions.

## Supplementary Materials

The supplementary materials are available online at https://www.mdpi.com/2072-6643/13/3/930/s1.
