# Supplementary material for: Lifestyle Changes and Determinants of Children’s and Adolescents’ Body Weight Increase during the First COVID-19 Lockdown in Greece: The COV-EAT Study
Source: Nutrients. 2021 Mar 13;13(3):930. doi: 10.3390/nu13030930 (PMC7998995; doi:10.3390/nu13030930)
Supplement: Supplementary file 1 [file nutrients-13-00930-s001.pdf]

Questionnaire about the eating habits of children and adolescents during the coronavirus pandemic (Covid-19).

The questionnaire will be open during the period 30/04/2020 - 24/05/2020.

Thank you in advance for your participation!

* Required

1. Consent form to participate in the study. *

- [ ] I declare that I am aware that the data in this questionnaire are confidential and are used anonymously for statistical processing and scientific publication.
- [ ] I do not wish to participate in this research.

Section 1: *Demographics.* *(In this section the questions concern both parents and children.)*

2. Date of Completion of the Questionnaire (day / month / year] *

____

3. Who completes the questionnaire? *

- [ ] Father
- [ ] Mother
- [ ] Other

4. Permanent Residence (Note City / Village)*

____

5. Prefecture of Permanent Residence *

____

	Father	Mother
Primary School	☐	☐
Junior High School	☐	☐
High School	☐	☐
Post High School Education (Vocational Training Institute)	☐	☐
Higher Education	☐	☐
Post Graduate Studies (MSc/PHD)	☐	☐

6. Education Level*

7. Occupational Status (Before Confinement) *

	Father	Mother
Working	☐	☐
Unemployed	☐	☐
Retired	☐	☐

8. If there was a change in the father's employment status during the incarceration, please indicate what kind of change was made: *

☐	No change
☐	I worked regular hours, as before the closure, but from home
☐	My working hours have been increased
☐	My working hours have been reduced
☐	I got a special purpose leave

☐	I became unemployed
☐	Other:………………………………….

9. If there was a change in the mother's occupational status during the confinement, please indicate what kind of change was made: *

☐	No change
☐	I worked regular hours, as before the closure, but from home
☐	My working hours have been increased
☐	My working hours have been reduced
☐	I got a special purpose leave
☐	I became unemployed
☐	Other:………………………………….

10. Marital Status*

☐	Married
☐	Divorced
☐	Widower/widow
☐	Single
☐	Other:………………………………….

11. Father's age*

____

12. Mother's age*

13. How many children do you have (note the number)? *

Important Note: **PLEASE NOTE THAT YOUR ANSWERS CONCERN ONLY ONE CHILD AGED 2-18**

14. Indicate if your child remained at home before confinement. * (For example if it is too young and you did not send it to school.)

- Yes, my child was staying at home before the confinement
- No , my child went to school.

15. Child's date of birth (day /month /year)*

16. Child's sex *

- Boy
- Girl

Section 2: Questions concerning parents/guardians.

17. How often do you cook? *	Before Confinement	After Confinement
Daily	☐	☐

5-6 times per week	☐	☐
3-4 times per week	☐	☐
1-2 times per week	☐	☐
1-3 times per month	☐	☐
Not at all/Never	☐	☐

18. Who usually cooks at home? *(You can choose more than one answers.)	Before Confinement	After Confinement
Mother	☐	☐
Father	☐	☐
Grandparents	☐	☐
Relative	☐	☐
Friends	☐	☐
Housekeeper	☐	☐

19. Which main meals did you eat / consume during the day? *(The main meals of the day are breakfast, lunch and dinner. Select all the valid answers).

	None	Breakfast	Lunch	Dinner
Before Confinement	☐	☐	☐	☐
After Confinement	☐	☐	☐	☐

20. How many snacks did you eat / consume per day? * (By snack we mean the small meals that we usually consume between the main meals, eg in the afternoon, before bed, etc.).

____

21. Have you increased snacking during confinement? *

☐	Not at all
☐	Very little
☐	A little
☐	Considerably
☐	Immensely

22. Which is/are the main reason/reasons for increasing snacking? * (You can check more than one answers.)

☐	I haven't increased snacking
☐	I feel bored
☐	I'm stressed
☐	Lack of sleep
☐	Hunger
☐	To boost my immune system
☐	Other:........................

23. What kind of food do you prefer for snacking?* (You can choose more than one answers.)

Salty snacks (potato chips, pop corns, crackers etc)

Sweets (chocolate, biscuits, cakes, ice cream etc)

Fruit /vegetables

- [ ] Dairy (milk ,cheese, yoghurt)
- [ ] Nuts
- [ ] Cereals/cereal bars/bread/breadsticks
- [ ] Cold cuts (turkey ,ham, salami etc)
- [ ] Soft drinks

24. How often do you order fast food?*

	Before Confinement	After Confinement
Daily	☐	☐
5-6 times per week	☐	☐
3-4 times per week	☐	☐
1-2 times per week	☐	☐
1-3 times per month	☐	☐
Not at all/Never	☐	☐

25. Are you interested in healthy eating?*

Yes ○

No ○

26. If yes, which is the main source of your information?

☐	Internet
☐	Newspapers/Magazines
☐	TV
☐	Dietitian/Nutritionist

☐ Other:……………………….

27. Are you trying to lose weight this period? *

Yes ◯

No ◯

28. Are you following a diet plan?*

Yes ◯

No ◯

29. If yes, when did you begin?

Before ◯ confinement

◯ During confinement

30.If you are following a diet plan where did you get it from?

Dietitian/Nutritionist

Doctor

Trainer

- Friend
- Internet
- Newspaper/magazine
- Other:..............................

31. Please note the father's weight (Weight in kg)

____

32. Please note the father's height (Height in cm)

____

33. Please note the mother's weight (Weight in kg)

____

34. Please note the mother's height.(Height in cm)

____

IMPORTANT NOTE: Your answers are concerning only one of your children aged 2-18

35. Please note your child's weight *(Weight in kg)

____

36. Please note your child's height. *(Height in cm)

____

37. Has your child's weight changed during the confinement*

- [ ] It didn't change

☐	It increased
☐	It decreased
☐	I don't know

38. If your child's weight was increased please note the number of kilos increased

____

39. Which main meals does your child consume?* (The main meals of the day are breakfast, lunch and dinner. You can write down more than one answer)

	None	Breakfast	Lunch	Dinner
Before Confinement	☐	☐	☐	☐
After Confinement	☐	☐	☐	☐

40. How often does your child consume breakfast?*

	Before Confinement	After Confinement
Daily	☐	☐
5-6 times per week	☐	☐
3-4 times per week	☐	☐
1-2 times per week	☐	☐
1-3 times per month	☐	☐
Not at all/Never	☐	☐

41. How many snacks does your child eat / consume per day? * (By snack we mean the snacks that we usually consume between the main meals, eg tithe, afternoon, bedtime, etc.)

	None	1	2	3	≥4
Before Confinement	☐	☐	☐	☐	☐

	None	1	2	3	≥4
After Confinement	☐	☐	☐	☐	☐

42. How often does your child eat fast food? * (By fast food we mean for example pizza, skewers, burgers, sandwiches etc.)

	Before Confinement	After Confinement
Daily	☐	☐
5-6 times per week	☐	☐
3-4 times per week	☐	☐
1-2 times per week	☐	☐
1-3 times per month	☐	☐
Not at all/Never	☐	☐

43. How often does your child eat fruits?

	Before Confinement	After Confinement
Daily	☐	☐
5-6 times per week	☐	☐
3-4 times per week	☐	☐
1-2 times per week	☐	☐
1-3 times per month	☐	☐
Not at all/Never	☐	☐

44.How many fruits per day, does your child consume? *

	None	1	2	3	4	>4
Before Confinement	☐	☐	☐	☐	☐	☐
After Confinement	☐	☐	☐	☐	☐	☐

45. How often does your child consume pre-packed fruit juices?* (When we say packaged juices we mean commercial juices with or without sugar.)

	Before Confinement	After Confinement
Daily	☐	☐
5-6 times per week	☐	☐
3-4 times per week	☐	☐
1-2 times per week	☐	☐
1-3 times per month	☐	☐
Not at all/Never	☐	☐

46.How many glasses of pre-packed fruit juice does your child consume on a daily basis? *
(One glass equals 250ml.)

	None	1	2	3	>3
Before Confinement	☐	☐	☐	☐	☐
After Confinement	☐	☐	☐	☐	☐

47.How often does your child consume fresh fruit juices? * (from fresh fruit with or without fiber, without added sugar.)

	Before Confinement	After Confinement
Daily	☐	☐
5-6 times per week	☐	☐
3-4 times per week	☐	☐

1-2 times per week	☐	☐
1-3 times per month	☐	☐
Not at all/Never	☐	☐

48. How many glasses of freshly squeezed fruit juice per day does your child consume?* (1 glass equals 250 ml.)

	None	1	2	3	>3
Before Confinement	☐	☐	☐	☐	☐
After Confinement	☐	☐	☐	☐	☐

49.How often does your child consume vegetables (cooked or raw)?

	Before Confinement	After Confinement
Daily	☐	☐
5-6 times per week	☐	☐
3-4 times per week	☐	☐
1-2 times per week	☐	☐
1-3 times per month	☐	☐
Not at all/Never	☐	☐

50.How many vegetables does your child consume per day? * (1 portion of vegetables is 1 cup raw vegetables and half a cup of boiled or grilled vegetables.)

	None	1	2	3	4	>4
Before Confinement	☐	☐	☐	☐	☐	☐
After Confinement	☐	☐	☐	☐	☐	☐

51. How often does your child consume dairy products?* (With the term dairy we mean milk ,yoghurt ,cheese)

	Before Confinement	After Confinement
Daily	☐	☐
5-6 times per week	☐	☐
3-4 times per week	☐	☐
1-2 times per week	☐	☐
1-3 times per month	☐	☐
Not at all/Never	☐	☐

52. How many portions of dairy products does your child consume per day?* {1 portion of dairy is 1 cup of milk (240 ml) or 1 cup of yogurt (150 g) or 1 matchbox of cheese (30 g)}

	None	1	2	3	4	>4
Before Confinement	☐	☐	☐	☐	☐	☐
After Confinement	☐	☐	☐	☐	☐	☐

53. How often does your child consume red meat? * (By red meat we mean beef, lamb, goat, pork etc.)

	Before Confinement	After Confinement
Daily	☐	☐
5-6 times per week	☐	☐
3-4 times per week	☐	☐
1-2 times per week	☐	☐
1-3 times per month	☐	☐
Not at all/Never	☐	☐

54. How often does your child consume poultry?* (By poultry we mean chicken, turkey, rabbit etc.)

	Before Confinement	After Confinement
Daily	☐	☐
5-6 times per week	☐	☐
3-4 times per week	☐	☐
1-2 times per week	☐	☐
1-3 times per month	☐	☐
Not at all/Never	☐	☐

55. How often does your child consume fish?*

	Before Confinement	After Confinement
Daily	☐	☐
5-6 times per week	☐	☐
3-4 times per week	☐	☐
1-2 times per week	☐	☐
1-3 times per month	☐	☐
Not at all/Never	☐	☐

56. How often does your child consume pasta, potatoes, rice?

	Before Confinement	After Confinement
Daily	☐	☐
5-6 times per week	☐	☐
3-4 times per week	☐	☐
1-2 times per week	☐	☐
1-3 times per month	☐	☐
Not at all/Never	☐	☐

57. How often does your child consume legumes? * (By legumes we mean beans, chickpeas, lentils, fava beans, broad beans etc.)

	Before Confinement	After Confinement
Daily	☐	☐
5-6 times per week	☐	☐
3-4 times per week	☐	☐
1-2 times per week	☐	☐
1-3 times per month	☐	☐
Not at all/Never	☐	☐

58. How often does your child consume homemade sweets? * (By homemade sweets we mean sweets that are made at home eg. cakes, biscuits, rice pudding, creams etc.)

	Before Confinement	After Confinement
Daily	☐	☐
5-6 times per week	☐	☐
3-4 times per week	☐	☐
1-2 times per week	☐	☐
1-3 times per month	☐	☐
Not at all/Never	☐	☐

59. How often does your child consume commercially available sweets? * (By commercially available sweets we mean sweets that can be found at supermarkets, confectioneries, bakeries, etc. eg. desserts with sugar, chocolate, milk-based desserts, cakes, croissants, cookies, pastries, ice creams etc.)

	Before Confinement	After Confinement
Daily	☐	☐
5-6 times per week	☐	☐
3-4 times per week	☐	☐
1-2 times per week	☐	☐
1-3 times per month	☐	☐

Not at all/Never	☐	☐

60. How often does your child consume salty snacks? * (By salty snacks we mean chips, savory cookies / crackers, pies such as cheese pies, ham and cheese pies, spinach pies etc.)

	Before Confinement	After Confinement
Daily	☐	☐
5-6 times per week	☐	☐
3-4 times per week	☐	☐
1-2 times per week	☐	☐
1-3 times per month	☐	☐
Not at all/Never	☐	☐

61. How often does your child consume soft drinks? * (We mean packaged soft drinks / energy drinks with added sugar.)

	Before Confinement	After Confinement
Daily	☐	☐
5-6 times per week	☐	☐
3-4 times per week	☐	☐
1-2 times per week	☐	☐
1-3 times per month	☐	☐
Not at all/Never	☐	☐

62. Is your child taking any vitamin / mineral supplements? *(You can choose more than one answers)

	Before Confinement	After Confinement
No supplements	☐	☐
Yes, a multivitamin	☐	☐

	Before Confinement	After Confinement
Yes, vitamin D	☐	☐
Yes, vitamin C	☐	☐
Yes, fatty acids	☐	☐
Another supplement	☐	☐

63. If your child was receiving vitamin D, determine in what form?

- Drops
- Spray
- Tablet
-
- Capsule

Other:………

64. Approximately how much time (in hours) per day does your child spend on screen activities? * (By screen activities we mean the hours out of school on TV, DVD, PC, tablet, smartphone, video games.)

	Before Confinement	After Confinement
Not at all	☐	☐
Less than one hour	☐	☐
More or equal with 1 hour and less than 2 hours	☐	☐
More or equal with 2 hours and less than 3 hours	☐	☐
More than 3 hours	☐	☐

	Before Confinement	After Confinement

< 8 hours	☐	☐
8-10 hours	☐	☐
>10 hours	☐	☐

65. How many hours does your child sleep at night? *

	Before Confinement	After Confinement
< 8 hours	☐	☐
8 – 10 hours	☐	☐
> 10 hours	☐	☐

66. Did your child's physical activity change during the confinement compared to before the confinement? * (By physical activity we mean participation in activities such as walking, running, cycling, exercise, etc.)

- ◯ It didn't change
- ◯ It increased
- It ◯ decreased
- I ◯ don't know /I don't answer

67. Did/Does your child follow a specific diet plan by a specialist? *

	Before Confinement	After Confinement
YES	☐	☐
NO	☐	☐

68.During the confinement, was your child diagnosed with any medical condition? *

- ◯ Yes
- ◯ No

69.If yes please note the medical condition.

70. Please note your e-mail if you wish
